# The complete mitogenome of *Achalarus lyciades* (Lepidoptera: Hesperiidae)

**DOI:** 10.1080/23802359.2016.1197070

**Published:** 2016-08-30

**Authors:** Jinhui Shen, Qian Cong, Nick V. Grishin

**Affiliations:** a Departments of Biophysics and Biochemistry, University of Texas Southwestern Medical Center, Dallas, TX, USA;; b Howard Hughes Medical Institute, University of Texas Southwestern Medical Center, Dallas, TX, USA

**Keywords:** Coeliadinae, eudaminae, lobocla, next-generation sequencing, phylogeny

## Abstract

We obtained a complete mitochondrial genome of a skipper butterfly *Achalarus lyciades* (Hesperiidae, Eudaminae) from next generation sequencing reads. The 15,612 bp mitogenome covers 13 protein-coding genes (PCGs), 22 transfer RNA genes (tRNAs), 2 ribosomal RNA genes (rRNAs) and an A + T rich region. Its gene order is typical for mitogenomes of Lepidoptera. Phylogenetic analysis placed *A. lyciades* as a sister to *Lobocla bifasciatus*, the only other Eudaminae with available mitogenome.

The Hoary Edge (*Achalarus lyciades*) is a skipper butterfly (family Hesperiidae, subfamily Eudaminae) widely distributed over the eastern United States. Its English name comes from a large whitish patch beneath the hindwing near the edge. To better understand the phylogeny of Hesperiidae, we sequenced, assembled and annotated the complete mitogenome of *A. lyciades* from the male voucher NVG-3311 collected in USA: Texas, Sabine Co., Sabine National Forest, 1 mi south of Fairmount, near Fox Hunters’ Hill, GPS 31.185394, −93.72992 on 12 April 2015. The body was stored in *RNAlater* solution and wings preserved to be deposited in the National Museum of Natural History, Smithsonian Institution, WA. Methods for genomic DNA extraction, library construction, next-generation sequencing and computational procedures followed those we reported previously (Shen et al. 2015; Cong et al. 2016a,b; Cong & Grishin 2016). Using mitogenome of *Choaspes benjaminii* as a reference to “bait” sequence reads, about 2.9% of reads (4,732,966 out of 163,971,978) were extracted by MITObim (Hahn et al. 2013) from the 250 bp library, followed by 15 mer JELLYFISH (Marcais & Kingsford 2011) counting and QUAKE (Kelley et al. 2010) error correction to exclude frequency lower than 1000 reads (Cong & Grishin 2016). The corrected reads were assembled denovo with Platanus. The 5' and 3' end of the 15491 bp Platanus assembly was inspected and extended manually to complete the circular structure (Cong & Grishin 2016). The genome sequence was annotated using the MITOS web server (Bernt et al. 2013), the predictions were manually curated using other published skipper mitogenomes as references. The assembly quality was assessed by reads mapping and sequence multiple alignment of the PCGs, tRNAs and rRNAs genes with published Hesperiidae mitogenomes for consistency checking.

The complete mitogenome of *A. lyciades* is 15,613 bp in length (Genbank: KX249739) and is AT rich, with a base composition of 40.5% A, 41.4% T, 7.3% G and 10.8% C. It retains the typical insect mitogenome gene set, including 13 PCGs (ND1-6, COX1-3, ND4L, ATP8, ATP6 and CYTB), 22 tRNA genes (two for serine and leucine and one for each of the rest amino acids), 2 ribosomal RNAs (rrnL and rrnS) and an A + T rich D-loop control region. In many Lepidoptera mitogenomes, COX1 gene starts from the codon CGA (Kim et al. 2009), however, in *A. lyciades*, all PCGs including COX1 use the typical start codon ATN. COX1, COX2 and ND4 have an incomplete stop codon T, and a complete TAA codon is likely formed during mRNA maturation (Ojala et al. 1981; Boore 1999). The length of tRNAs ranges from 61 to 73 bp. The size of the two rRNAs are 1378 and 778 bp, respectively. A 452 bp A + T rich region connects rrnS and tRNA-Met.

To phylogenetically place *A. lyciades* within Hesperiidae with available mitogenomes (Hao et al. 2012; Wang et al. 2013; Kim et al. 2014; Wang et al. 2014; Shao et al. 2015; Shen et al. 2015; Wang et al. 2015; Cong & Grishin 2016; Jiang et al. 2016), we constructed RAxML (Stamatakis 2006) maximum likelihood tree rooted with *Papilio glaucus* (Papilionidae) mitogenome (Shen et al. 2015) (Figure 1). Sister relationship of *A. lyciades* and *Lobocla bifasciatus* is strongly supported, in agreement with the placement of *Lobocla* as a sole Old Word genus in the mostly New World subfamily Eudaminae (Warren et al. 2008; Warren et al. 2009; Yuan et al. 2015). The tree topology is largely consistent with previous phylogenetic studies (Warren et al. 2008; Warren et al. 2009; Yuan et al. 2015): Coeliadinae are the sister to all other Hesperiidae; among taxa with available mitogenomes, Tagiadini and Celaenorrhini are sisters, Heteropterinae are the sister to Hesperiinae within which Aeromachini is the sister to the rest. However, bootstrap on mitogenomes is insufficient to support monophyly of Pyrginae, and the topology within the crown Hesperiinae group deviates from that reported previously and needs to be investigated further. In conclusion, the complete mitogenome of *A. lyciades* is the first one for a New Word representative of subfamily Eudaminae essential for further studies of Lepidoptera.

**Figure 1.**
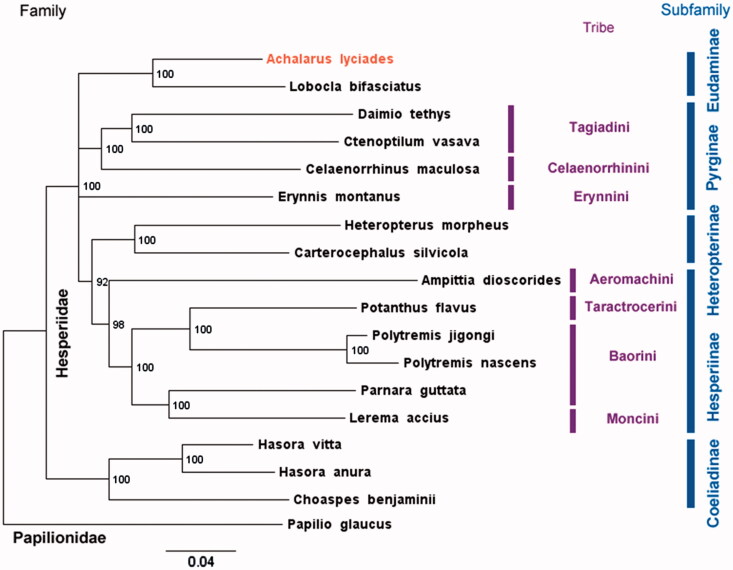
Maximum likelihood tree of complete mitogenomes of 18 Hesperiidae species rooted with *Papilio glaucus* (Papilionidae). *Achalarus lyciades* with mitogenome reported here is shown first. Numbers by the nodes show bootstrap support values and branches with bootstrap less than 50% are collapsed. GenBank accessions for sequences are: *Ampittia dioscorides* KM102732.1; *Celaenorrhinus maculosa* NC_022853.1; *Daimio Tethys* NC_024648.1; *Erynnis montanus* NC_021427.1; *Hasora anura* NC_027263.1; *Hasora vitta* NC_027170.1; *Heteropterus morpheus* NC_028506.1 *Choaspes benjaminii* NC_024647.1; *Lerema accius* NC_029826.1; *Lobocla bifasciatus* NC_024649.1; *Carterocephalus silvicola* NC_024646.1; *Polytremis jigongi* NC_026990.1; *Polytremis nascens* NC_026228.1; *Potanthus flavus* NC_024650.1; *Parnara guttata* NC_029136.1; *Ctenoptilum vasava* NC_016704.1; *Papilio glaucus* NC_027252.

## References

[CIT0001] Bernt M, Donath A, Jühling F, Externbrink F, Florentz C, Fritzsch G, Pütz J, Middendorf M, Stadler PF. 2013 MITOS: improved de novo metazoan mitochondrial genome annotation. Mol Phylogenet Evol. 69:313–319. 22982435 10.1016/j.ympev.2012.08.023

[CIT0002] Boore JL. 1999 Animal mitochondrial genomes. Nucleic Acids Res. 27:1767–1780. 10101183 10.1093/nar/27.8.1767 PMC148383

[CIT0003] Cong Q, Shen J, Borek D, Robbins RK, Otwinowski Z, Grishin NV. 2016a Complete genomes of hairstreak butterflies, their speciation, and nucleo-mitochondrial incongruence. Sci Rep. 6:24863. 27120974 10.1038/srep24863 PMC4848470

[CIT0004] Cong Q, Shen J, Warren AD, Borek D, Otwinowski Z, Grishin NV. 2016b Speciation in cloudless sulphurs gleaned from complete genomes. Genome Biol Evol. 8:915–931. doi:10.1093/gbe/evw045 26951782 PMC4894063

[CIT0005] Cong Q, Grishin NV. 2016 The complete mitochondrial genome of Lerema accius and its phylogenetic implications. Peer J. 4:e1546. 26788426 10.7717/peerj.1546 PMC4715447

[CIT0006] Hahn C, Bachmann L, Chevreux B. 2013 Reconstructing mitochondrial genomes directly from genomic next-generation sequencing reads – a baiting and iterative mapping approach. Nucleic Acids Research. 41:e129. 23661685 10.1093/nar/gkt371 PMC3711436

[CIT0007] Hao J, Sun Q, Zhao H, Sun X, Gai Y, Yang Q. 2012 The complete mitochondrial genome of *Ctenoptilum vasava* (Lepidoptera: hesperiidae: pyrginae) and its phylogenetic implication. Compar Func Genomics. 2012:328049. 10.1155/2012/328049 PMC3335176 22577351

[CIT0008] Jiang W, Zhu J, Yang Q, Zhao H, Chen M, He H, Yu W. 2016 Complete mitochondrial DNA genome of *Polytremis nascens* (Lepidoptera: Hesperiidae). Mitochondrial DNA A DNA MappSeq Anal. 27:3131–3132. 25690054 10.3109/19401736.2015.1007298

[CIT0009] Kelley DR, Schatz MC, Salzberg SL. 2010 Quake: quality-aware detection and correction of sequencing errors. Genome Biol. 11:R116. 21114842 10.1186/gb-2010-11-11-r116 PMC3156955

[CIT0010] Kim MI, Baek JY, Kim MJ, Jeong HC, Kim KG, Bae CH, Han YS, Jin BR, Kim I. 2009 Complete nucleotide sequence and organization of the mitogenome of the red-spotted apollo butterfly, Parnassius bremeri (Lepidoptera: papilionidae) and comparison with other lepidopteran insects. Mol Cells. 28:347–363. 19823774 10.1007/s10059-009-0129-5

[CIT0011] Kim MJ, Wang AR, Park JS, Kim I. 2014 Complete mitochondrial genomes of five skippers (Lepidoptera: Hesperiidae) and phylogenetic reconstruction of Lepidoptera. Gene. 549:97–112. 25058696 10.1016/j.gene.2014.07.052

[CIT0012] Marcais G, Kingsford C. 2011 A fast, lock-free approach for efficient parallel counting of occurrences of k-mers. Bioinformatics. 27:764–770. 21217122 10.1093/bioinformatics/btr011 PMC3051319

[CIT0013] Ojala D, Montoya J, Attardi G. 1981 tRNA punctuation model of RNA processing in human mitochondria. Nature. 290:470–474. 7219536 10.1038/290470a0

[CIT0014] Shao L, Sun Q, Hao J. 2015 The complete mitochondrial genome of Parara guttata (Lepidoptera: Hesperiidae). Mitochondrial DNA. 26:724–725. 24460163 10.3109/19401736.2013.845759

[CIT0015] Shen J, Cong Q, Grishin NV. 2015 The complete mitochondrial genome of Papilio glaucus and its phylogenetic implications. Meta Gene. 5:68–83. 26106582 10.1016/j.mgene.2015.05.002 PMC4475787

[CIT0016] Stamatakis A. 2006 RAxML-VI-HPC: maximum likelihood-based phylogenetic analyses with thousands of taxa and mixed models. Bioinformatics. 22:2688–2690. 16928733 10.1093/bioinformatics/btl446

[CIT0017] Wang AR, Jeong HC, Han YS, Kim I. 2014 The complete mitochondrial genome of the mountainous duskywing, Erynnis montanus (Lepidoptera: hesperiidae): a new gene arrangement in Lepidoptera. Mitochondrial DNA. 25:93–94. 23586336 10.3109/19401736.2013.784752

[CIT0018] Wang J, James John Y, Xuan S, Cao T, Yuan X. 2015 The complete mitochondrial genome of the butterfly Hasora anura (Lepidoptera: hesperiidae). Mitochondrial DNA. 1–2. 26466192 10.3109/19401736.2015.1089543

[CIT0019] Wang K, Hao J, Zhao H. 2013 Characterization of complete mitochondrial genome of the skipper butterfly, Celaenorrhinus maculosus (Lepidoptera: hesperiidae). Mitochondrial DNA. 26:690–691. 24102602 10.3109/19401736.2013.840610

[CIT0020] Warren AD, Ogawa JR, Brower AVZ. 2008 Phylogenetic relationships of subfamilies and circumscription of tribes in the family Hesperiidae (Lepidoptera: hesperioidea). Cladistics. 24:642–676.

[CIT0021] Warren AD, Ogawa JR, Brower AVZ. 2009 Revised classification of the family Hesperiidae (Lepidoptera: hesperioidea) based on combined molecular and morphological data. Syst Entomol. 34:467–523.

[CIT0022] Yuan X, Gao K, Yuan F, Wang P, Zhang Y. 2015 Phylogenetic relationships of subfamilies in the family Hesperiidae (Lepidoptera: hesperioidea) from China. Sci Rep. 5:11140. 26059470 10.1038/srep11140 PMC4461911

